# Supplementary material for: Direct 4D printing of ceramics driven by hydrogel dehydration
Source: Nat Commun. 2024 Jan 26;15:758. doi: 10.1038/s41467-024-45039-y (PMC10810896; doi:10.1038/s41467-024-45039-y)
Supplement: Supplementary file 1 — Supplementary Information [file 41467_2024_45039_MOESM1_ESM.pdf]

Supplementary Information for

Direct 4D printing of ceramics driven by hydrogel dehydration

Rong Wang[1,2,+], Chao Yuan[3,+,*], Jianxiang Cheng[1,2,+], Xiangnan He[1,2], Haitao Ye[1,2,4], Bingcong Jian[1,2], Honggeng Li[1,2], Jiaming Bai[2], Qi Ge[1,2,*]

[1]Shenzhen Key Laboratory of Soft Mechanics & Smart Manufacturing, Southern University of Science and Technology, Shenzhen 518055, China.

[2]Department of Mechanical and Energy Engineering, Southern University of Science and Technology, Shenzhen 518055, China.

[3]State Key Laboratory for Strength and Vibration of Mechanical Structures, Department of Engineering Mechanics, Xi'an Jiaotong University, Xi'an 710049, China.

[4]Department of Mechanical Engineering, City University of Hong Kong, Kowloon, Hong Kong SAR, China.

[+] These authors contributed equally to this work.

[*] Corresponding author. E-mail: chao_yuan@xjtu.edu.cn; geq@sustech.edu.cn

Supplementary Note 1. Characterization of slurry uniformity

The ceramic slurry is composed of photosensitive resin (BA and PEGDA), ceramic powder, dispersant and photoinitiator. The dispersant (KOS 110, Guangzhou Kangoushuang Trade Co., Ltd., China) enables ceramic particles to be better dispersed in the resin. In order to break up agglomerated ceramic particles and ensure that these particles are dispersed uniformly enough in the resin, we use ball milling to mix them. For comparison, we also prepared ceramic slurry using a planetary mixer (ZYMC-180HV, Shenzhen ZYE Technology Co., Ltd., China). Supplementary Fig. 1 compares the ceramic slurry prepared using a ball mill with that prepared using a planetary mixer. As shown in Supplementary Fig. 1a and 1c, we take a droplet of slurry onto a glass sheet and blow the droplet with compressed air to form a film. From the optical images in Supplementary Fig. 1b and 1d, we can see that the slurry prepared using the planetary mixer contains a large number of agglomerated ceramic particles with a size of tens of microns, while the slurry prepared using ball milling has no obvious particles. Therefore, the ball milling method can guarantee the ceramic slurry uniform and homogenous.

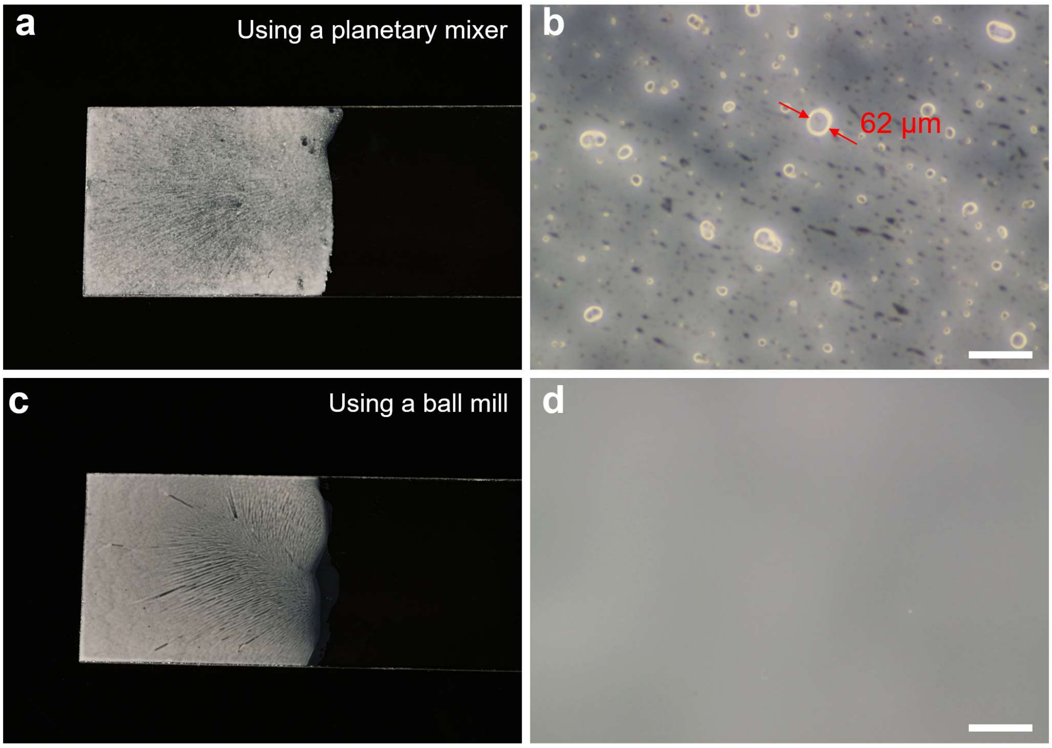

Supplementary Figure 1. Optical images of ceramic slurries prepared by different mixing methods. a,b, Using a planetary mixer. **c,d,** Using a ball mill. Scale bars, 200 μm.

Supplementary Note 2. Debinding and sintering processes

In order to develop an appropriate debinding process, we conducted thermogravimetry analysis (TGA) experiment. TGA (STA 449 F3, NETZSCH, Germany) was carried out in nitrogen with flow rate of 50 mL/min at a heating rate of 10 ℃/min. Supplementary Fig. 2a shows the thermogravimetry-derivative thermogravimetry (TG-DTG) curves of ceramic elastomer and dehydrated AP hydrogel. The TG curves indicate that the organics in ceramic elastomer and AP hydrogel mainly decompose at 300 ℃-500 ℃. The DTG curves indicate that the organics in ceramic elastomer and AP hydrogel decompose most violently at 398.3 ℃ and 423.8 ℃, respectively. Based on these results, we formulated the debinding process with multi-step heating strategy as shown in Supplementary Fig. 2b. The debinding process was carried out in argon using a tube furnace. When the temperature was lower than 300 ℃, a relatively fast heating rate of 1 ℃/min was adopted to save time. Then the samples were heated up to 400 ℃ for 2 h, 425 ℃ for 2 h, and 550 ℃ for 4 h, respectively. The heating rate was set as 0.25 ℃/min. This process ensured that the gas produced by the decomposition of organics could slowly escape from the inside of material without cracking or destroying the structure. After 4 h at 550 ℃, the debinding process was basically completed. Finally, the temperature was raised to 800 ℃ and held for 2 h. After that, the structure obtained enough holding strength to keep intact when it was transferred to a muffle furnace for sintering.

Supplementary Fig. 2c shows photographs of hydrogel-ceramic laminate after printing, dehydration and debinding, respectively. The AP hydrogel and ceramic elastomer have good interfacial strength, which ensures that the hydrogel-ceramic elastomer laminates can maintain the integrity of the structure during the dehydration and deformation processes without interface peeling. After debinding in argon, the hydrogel is removed along with the organics in ceramic elastomer, and the ceramic part remains. Supplementary Fig. 2d shows the sintering process. The sintering process was conducted in a muffle furnace with a maximum service temperature of 1700 ℃. The debinded ceramic sample was heated up to 1450 ℃ by multi-step heating and held for 2 h for sintering. The optimal sintering temperature is obtained by measuring the three-point bending strength of ceramic specimens after sintered at different temperatures. The specimens which exhibit a maximum flexural strength have the optimal sintering temperature.

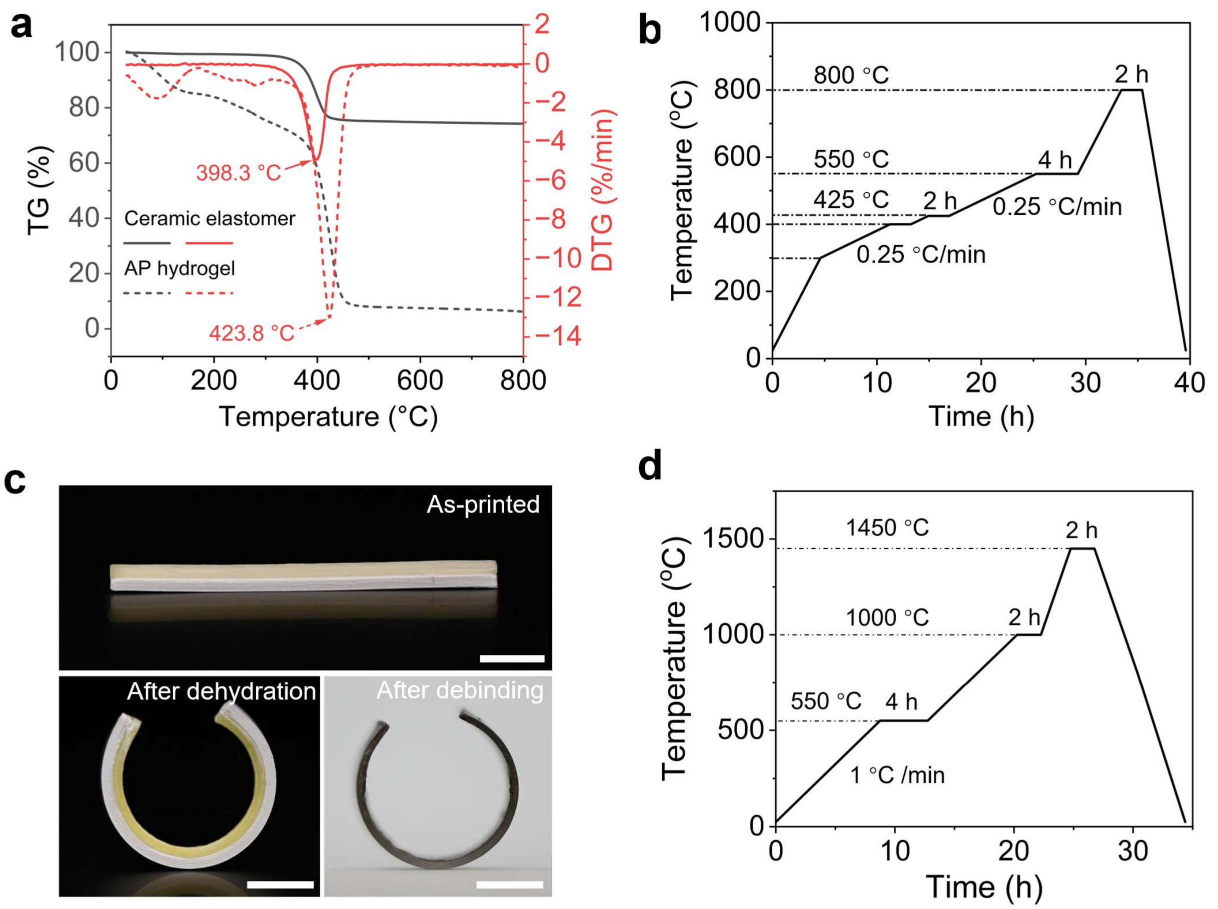

Supplementary Figure 2. Debinding and sintering processes. a, TG-DTG curves of ceramic elastomer and dehydrated AP hydrogel. Source data are provided as a Source Data file. **b,** The debinding process in argon. **c,** Photographs of hydrogel-ceramic laminate after printing, dehydration and debinding, respectively. Scale bars, 5 mm. **d,** The sintering process in air.

Supplementary Note 3. Three-point bending test

A series of ceramic specimens were printed and then sintered at different temperatures. We carried out three-point bending test using MTS machine (Model E45, MTS Systems Corporation, USA) equipped with three-point bending fixture. The loading rate was set as 0.5 mm/min. A typical force-displacement curve is shown in Supplementary Fig. 3a. The following equation is used to calculate the flexural strength σ_b:

$$\sigma_b = \frac{3PL}{2bh^2}$$

where P is the maximum force, L is the support span, b is the width of specimen, and h is the thickness of specimen. Supplementary Fig. 3b shows the flexural strength of ZrO_2 ceramic after sintered at different temperatures. The ceramic sintered at 1450 °C has the highest flexural strength (~800 MPa). Therefore, 1450 °C is the optimal sintering temperature for ZrO_2 ceramic in this work.

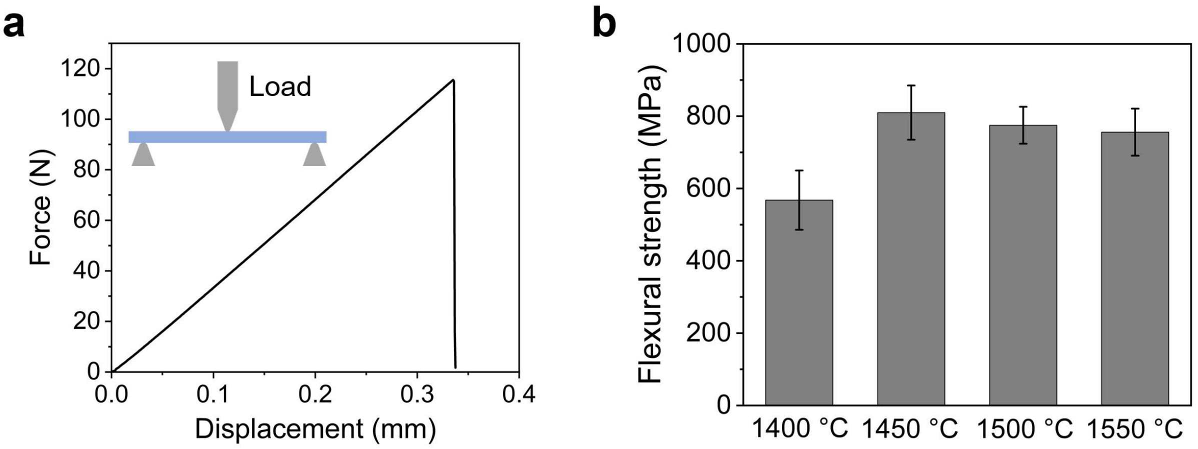

Supplementary Figure 3. Three-point bending test. a, Typical force-displacement plot of three-point bending test. The inset shows the illustration of three-point bending test. **b,** Flexural strength of ZrO_2 ceramic after sintering at different temperatures. The error bars represent the standard deviation of flexural strength. Source data are provided as a Source Data file.

Supplementary Note 4. Volume variation of ceramic layer after dehydration, debinding and sintering

Supplementary Fig. 4 shows the photographs of hydrogel-ceramic laminate at different stages. In the dehydration process, the ceramic elastomer layer occurs bending deformation, and its volume can be approximated as unchanged. During the debinding process, the organics in the ceramic elastomer are decomposed along with the dehydrated hydrogel, and the ceramic part remains. After debinding at 800 ℃, the entire ceramic structure has a linear shrinkage of ~3%, corresponding to a volume shrinkage of ~8.7%. After sintering at 1450 ℃, the ceramic has a linear shrinkage of 21.74%, corresponding to a volume shrinkage of ~52.1%.

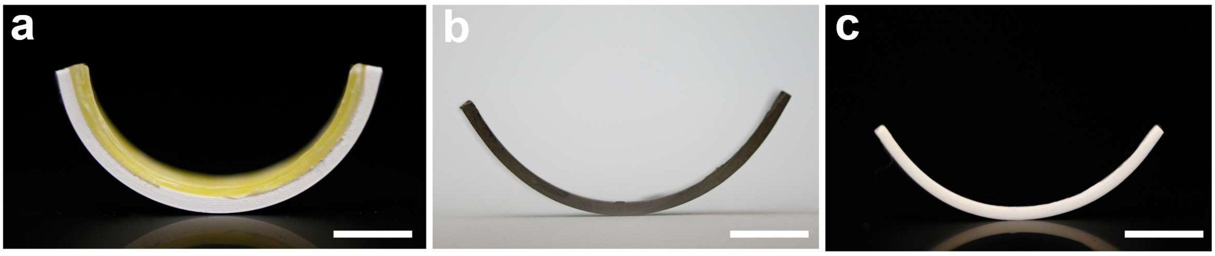

Supplementary Figure 4. Photographs of hydrogel-ceramic laminate at different stages. a, After dehydration. **b,** After debinding. **c,** After sintering. Scale bars, 5 mm.

Supplementary Note 5. Mechanical properties of ceramic elastomers

The PEGDA is used as crosslinker. The mechanical properties of the printed ceramic elastomer can be regulated by adjusting the content of PEGDA. Here, the PEGDA content refers to the mass fraction of PEGDA in the BA-PEGDA resin mixture. Supplementary Fig. 5 shows the Young's modulus and fracture strain of ceramic elastomer with different PEGDA contents. The ceramic elastomer with 1 wt.% PEGDA has a modulus of ~10 MPa and fracture stain of ~700%. The ceramic elastomer with 10 wt.% PEGDA has a modulus of ~18 MPa and fracture stain of ~40%.

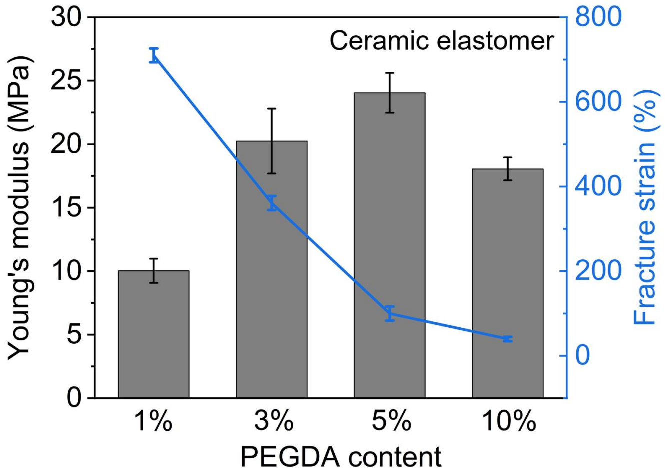

Supplementary Figure 5. Young's modulus and fracture strain of ceramic elastomer with different PEGDA contents. The error bars represent the standard deviation. Source data are provided as a Source Data file.

Supplementary Note 6. Changes in water content and bending curvature of hydrogel-ceramic laminate over time during the dehydration process

For the hydrogel-ceramic laminate with L of 30 mm, W of 5 mm, H_{hg} of 1mm and H_{ce} of 0.8 mm, Supplementary Fig. 6 shows the changes in water content and bending curvature of the laminate over time during dehydration. First, the laminate is dehydrated at room temperature (25 °C). After 7 h at room temperature, the remaining water content in the hydrogel is ~29% and the bending curvature of laminate is 0.160 mm^{-1}. Then, the laminate is placed in an 80 °C oven to continue the dehydration process. After dried at 80 °C for 4 h, the water content decreases to ~10% and the bending curvature is 0.194 mm^{-1}.

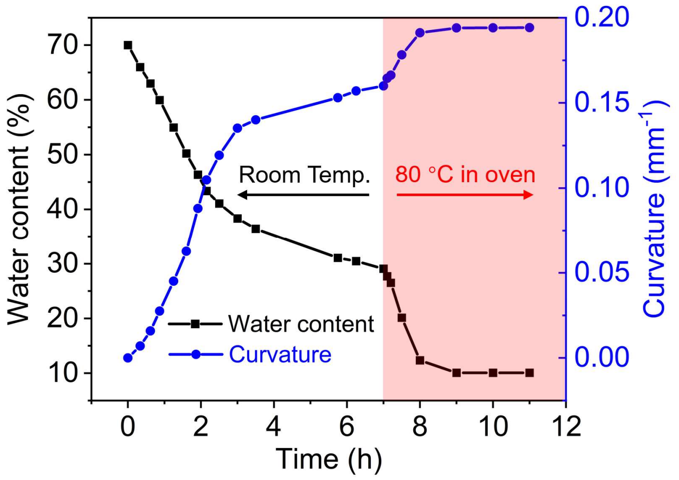

Supplementary Figure 6. Water content and bending curvature of hydrogel-ceramic laminate as a function of time during the dehydration process. Source data are provided as a Source Data file.

Supplementary Note 7. Phase-evolution based constitutive model for dehydrated hydrogel

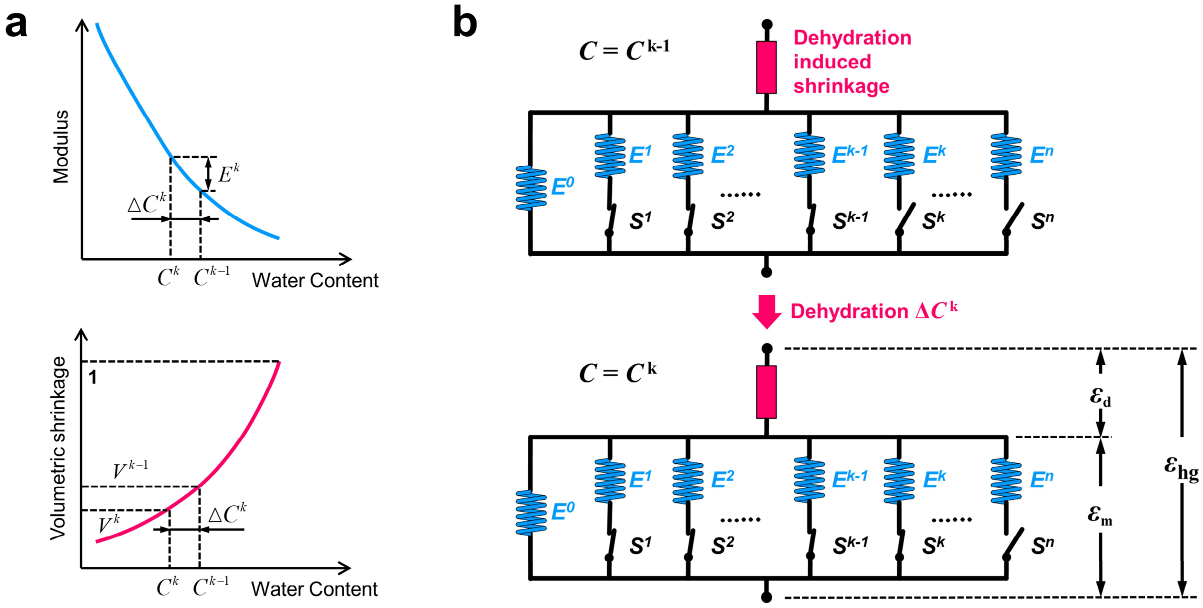

Supplementary Figure 7. Phase evolution model for dehydrated hydrogel. a, Schematic illustration of the modulus escalation and volumetric shrinkage during hydrogel dehydration. **b,** Schematic illustration of the phase formation process.

As demonstrated in Supplementary Fig. 7a, hydrogel dehydration leads to significant material stiffening and volume shrinkage. To model this stimuli-responsive behavior from the perspective of solid mechanics, we adopt the phase evolution approach and phenomenologically treat the dehydrated hydrogel as a multi-phase solid whose mechanical performance is cooperatively determined by all individual phases.

As shown in Supplementary Fig. 7b, a multi-branch spring-switch system consisting of one rubbery phase (E^0) and n glassy phases ($E^1 \sim E^n$) is schematically constructed to discretize the entire phase evolution process into n individual increments, each of which corresponds to a specific water content C . On the other hand, a volume change component is arranged in series with the multi-branch system to separate the dehydration induced shrinkage from the mechanical deformation. Following the tandem arrangement, the total strain of the hydrogel

$$\varepsilon_{\mathrm{hg}} = \varepsilon_{\mathrm{m}} + \varepsilon_{\mathrm{d}} , \tag{S1}$$

where ε_{m} and ε_{d} respectively denote the mechanical strain and dehydration-induced shrinkage

strain. At the rth increment, the dehydration strain $\varepsilon_{\text{d}}^{r}$ is

$$\varepsilon_{\text{d}}^{r} = \sum_{i=1}^{r} \Delta\varepsilon_{\text{d}}^{i} \,. \tag{S2}$$

where $\Delta\varepsilon_{\text{d}}^{i}$ represents the dehydration strain increment.

Our phase evolution adopts two basic assumptions. First, the rth phase is formed in a stress-free configuration and able to carry external loading after the rth increment. Second, at the rth increment, all r +1 phases (including the rubbery phase E^0) undergo identical mechanical strain increment $\Delta\varepsilon_{\text{m}}^{r}$ and the temporary elastic modulus of the hydrogel is calculated by

$$E_{\text{hg}}^{r} = \sum_{j=0}^{r} E^{j} \tag{S3}$$

where E^{j} represents the modulus of the jth glassy phase.

According to assumption 1, the hydrogel is fully occupied by the rubbery phase E^0 prior to dehydration ($C = C^0$). In this state, all switches ($S^1 \sim S^n$) in the multi-branch system maintain the "off" state and all external loading is carried by E^0.

As dehydration proceeds, the switch-controlled branches ($E^1 \sim E^n$) are sequentially activated to share the total stress. Consider a general state where k phases ($E^1 \sim E^{k\text{-}1}$) exist in the dehydrated hydrogel whose water content is reduced to $C^{k\text{-}1}$. By accumulating the stresses from the existing phases, the total stress σ_{hg}^{k-1} is obtained by

$$\sigma_{\text{hg}}^{k-1} = \sum_{i=0}^{k-1} E^{i} \varepsilon_{\text{m}}^{i,k-1} \tag{S4}$$

where $\varepsilon_{\text{m}}^{i,k-1}$ represents the mechanical strain of the ith phase in the $(k-1)^{\text{th}}$ increment.

After the water content decreases by ΔC^k, a new phase E^k is generated in the kth increment. According to assumption 2, all the existing k+1 phases ($E^1 \sim E^k$) experience the same mechanical strain increment $\Delta\varepsilon_{\text{m}}^{k}$. Moreover, because phase E^k is formed in a stress-free configuration, the updated total stress σ_{hg}^{k} is computed by

$$\sigma_{\text{hg}}^{k} = \sum_{i=0}^{k-1} E^{i} \left(\varepsilon_{\text{m}}^{i,k-1} + \Delta\varepsilon_{\text{m}}^{k}\right) + E^{k} \Delta\varepsilon_{\text{m}}^{k} \tag{S5}$$

Subtracting Eq. (S4) from Eq. (S5) yields the stress increment

$$\triangle\sigma_{\mathrm{hg}}^{k}=\sum_{i=0}^{k}E^{i}\triangle\varepsilon_{\mathrm{m}}^{k} \quad \text{(S6)}$$

Eq. (S6) provides a general form of the stress increment induced by phase generation. With Eq. (S1), we rewrite Eq. (S6) into

$$\triangle\sigma_{\mathrm{hg}}^{k}=\sum_{i=0}^{k}E^{i}\left(\triangle\varepsilon_{\mathrm{hg}}^{k}-\triangle\varepsilon_{\mathrm{d}}^{k}\right) \quad \text{(S7)}$$

where $\triangle\varepsilon_{\mathrm{hg}}^{k}$ and $\triangle\varepsilon_{\mathrm{d}}^{k}$ represent the increments of total strain and dehydration strain.

Supplementary Note 8. Deformation of the bent laminate

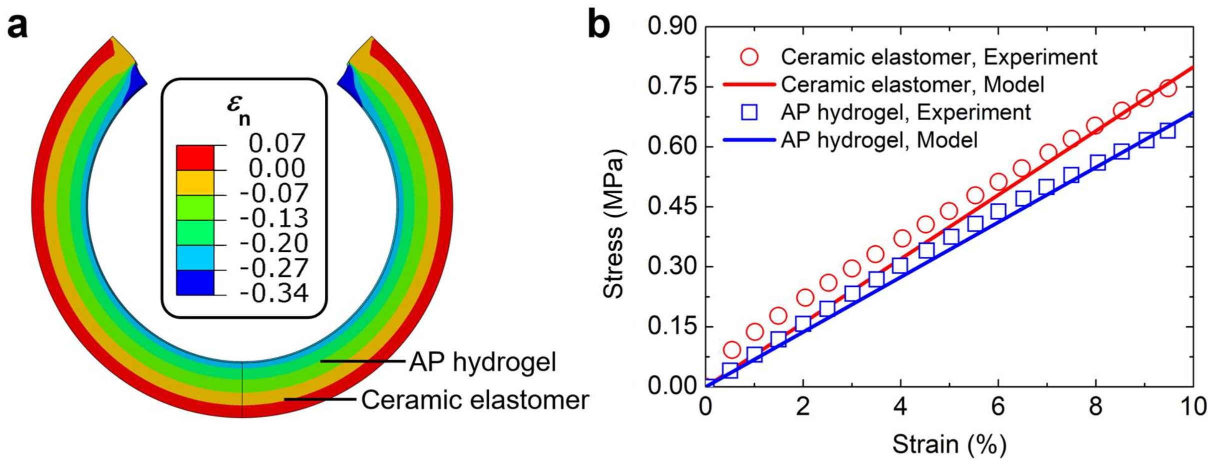

Supplementary Figure 8. Deformation of the bent laminate. a, Strain contour of the hydrogel-ceramic laminate after dehydration. The inner layer is AP hydrogel. The outer layer is ceramic elastomer. **b,** Comparison of the stress-strain curves between linear elastic model and experiment. Red: ceramic elastomer. Blue: AP hydrogel. Source data are provided as a Source Data file.

FE simulation result (Supplementary Fig. 8a) indicates that the maximum value of the tensile longitudinal strain (ε_n) within the dehydrated hydrogel-ceramic laminate is less than 10%. For convenience, we employ linear elastic model to describe the stress-strain relation. Supplementary Fig. 8b respectively compares the experimental and fitted stress-strain curves for AP hydrogel and ceramic elastomer. Good agreement indicates that linear elastic model is adequate to describe the mechanical performances of AP hydrogel and ceramic elastomer in this work.

Supplementary Note 9. Bilayer beam model for the hydrogel-ceramic laminate

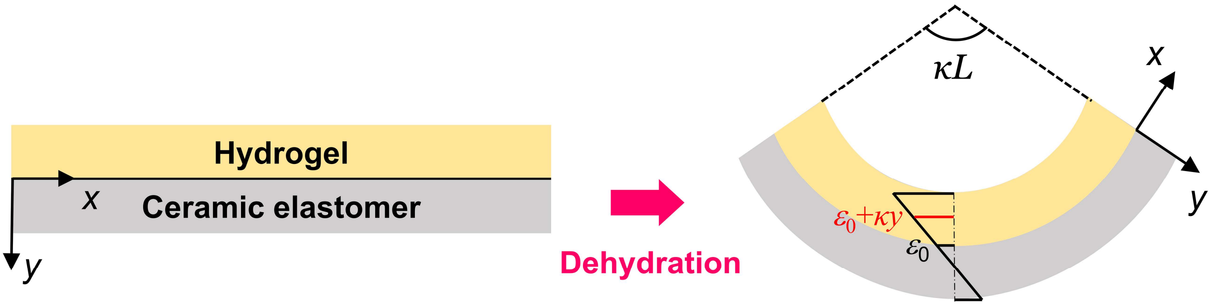

Supplementary Figure 9. Schematic illustration of the strain distribution along thickness of the bent hydrogel-ceramic laminate upon dehydration.

To assist the design of the hydrogel-ceramic laminate, the hydration-induced bending behavior is theoretically investigated. As shown in Supplementary Fig. 9, the bilayer laminate will spontaneously bend into an arc shape due to strain mismatch. According to the classical Euler-Bernoulli beam theory, the total strain increment of any material point along the cross section can be calculated by

$$\Delta\varepsilon^{i}=\begin{cases}\varepsilon_0^{i}+\kappa^{i}y, & i=1\\ \varepsilon_0^{i}+\kappa^{i}y-\left(\varepsilon_0^{i-1}+\kappa^{i-1}y\right), & i\geq 2\end{cases} \tag{S8}$$

where ε_0^{i} and κ^{i} respectively represent the boundary normal strain and bending curvature of the ith increment.

Since the ceramic elastomer maintains a moderate deformation upon bending, the stress of ceramic elastomer in the kth increment is

$$\sigma_{\text{ce}}^{k}=E_{\text{ce}}\left(\varepsilon_0^{k}+\kappa^{k}y\right) \tag{S9}$$

Herein, the total strain $\varepsilon_0^{k}+\kappa^{k}y$ is equivalent to the mechanical strain.

For the hydrogel, the stress incrementally updates with the declining water content *C*. By substituting Eq. (S8) into Eq. (S7) and accumulating the stress increments from 1 to *k*, we rewrite the stress of hydrogel into

$$\sigma_{\text{hg}}^{k}=\left(E^{0}+E^{1}\right)\left(\varepsilon_{0}^{1}+\kappa^{1}y-\Delta\varepsilon_{\text{d}}^{1}\right)+\left(\sum_{i=2}^{k}\sum_{j=0}^{i}E^{j}\varepsilon_{0}^{i}-\sum_{i=2}^{k}\sum_{j=0}^{i}E^{j}\varepsilon_{0}^{i-1}\right)$$
$$+\left(\sum_{i=2}^{k}\sum_{j=0}^{i}E^{j}\kappa^{i}y-\sum_{i=2}^{k}\sum_{j=0}^{i}E^{j}\kappa^{i-1}y\right)-\sum_{i=2}^{k}\sum_{j=0}^{i}E^{j}\Delta\varepsilon_{\text{d}}^{i} \tag{S10}$$

With some mathematical derivations, the last three terms in Eq. (S10) can be further simplified as

$$\sum_{i=2}^{k}\sum_{j=0}^{i}E^{j}\varepsilon_{0}^{i}-\sum_{i=2}^{k}\sum_{j=0}^{i}E^{j}\varepsilon_{0}^{i-1}=\sum_{j=0}^{k}E^{j}\varepsilon_{0}^{k}-\sum_{i=2}^{k}E^{i}\varepsilon_{0}^{i-1}-\left(E^{0}+E^{1}\right)\varepsilon_{0}^{1} \tag{S11}$$

$$\sum_{i=2}^{k}\sum_{j=0}^{i}E^{j}\kappa^{i}y-\sum_{i=2}^{k}\sum_{j=0}^{i}E^{j}\kappa^{i-1}y=\sum_{j=0}^{k}E^{j}\kappa^{k}y-\sum_{i=2}^{k}E^{i}\kappa^{i-1}y-\left(E^{0}+E^{1}\right)\kappa^{1}y \tag{S12}$$

$$\sum_{i=2}^{k}\sum_{j=0}^{i}E^{j}\Delta\varepsilon_{\text{d}}^{i}=\left(E^{0}+E^{1}\right)\sum_{i=2}^{k}\Delta\varepsilon_{\text{d}}^{i}+\sum_{j=2}^{k}E^{j}\sum_{i=j}^{k}\Delta\varepsilon_{\text{d}}^{i} \tag{S13}$$

By substituting Eq. (S2), (S3) and (S11)-(S13) into Eq. (S10), the total stress of the hydrogel at the kth increment is finally expressed by

$$\sigma_{\text{hg}}^{k}=E_{\text{hg}}^{k}\varepsilon_{0}^{k}+E_{\text{hg}}^{k}\kappa^{k}y-\sum_{i=2}^{k}\left(E_{\text{hg}}^{i}-E_{\text{hg}}^{i-1}\right)\varepsilon_{0}^{i-1}-\sum_{i=2}^{k}\left(E_{\text{hg}}^{i}-E_{\text{hg}}^{i-1}\right)\kappa^{i-1}y$$
$$-E_{\text{hg}}^{1}\varepsilon_{\text{d}}^{k}-\sum_{j=2}^{k}\left(E_{\text{hg}}^{j}-E_{\text{hg}}^{j-1}\right)\left(\varepsilon_{\text{d}}^{k}-\varepsilon_{\text{d}}^{j-1}\right) \tag{S14}$$

Since the bilayer laminate maintains a constraint-free state upon bending, the total external force and moment at any cross section are equal to 0. Thus, we have

$$\begin{cases}\sum F^{k}=\int_{0}^{H_{\text{ce}}}\sigma_{\text{ce}}^{k}dy+\int_{-H_{\text{hg}}^{k}}^{0}\sigma_{\text{hg}}^{k}dy=0\\ \sum M^{k}=\int_{0}^{H_{\text{ce}}}\sigma_{\text{ce}}^{k}ydy+\int_{-H_{\text{hg}}^{k}}^{0}\sigma_{\text{hg}}^{k}ydy=0\end{cases}. \tag{S15}$$

Herein we note that due to the existence of dehydration induced shrinkage, H_{hg}^{k} is required to be updated in each increment.

By substituting Eq. (S9) and (S14) into Eq. (S15), the boundary strain ε_{0}^{k} and bending curvature κ^{k} can be iteratively solved from

$$\begin{Bmatrix}\varepsilon_{0}^{k}\\ \kappa^{k}\end{Bmatrix}=\begin{bmatrix}A^{k} & B^{k}\\ B^{k} & D^{k}\end{bmatrix}^{-1}\begin{Bmatrix}N^{k}\\ Q^{k}\end{Bmatrix} \tag{S16}$$

where $A^k = E_{\mathrm{ce}} H_{\mathrm{ce}} + E_{\mathrm{hg}}^k H_{\mathrm{hg}}^k$,

$$B^k = \left[E_{\mathrm{hg}}^k \left(H_{\mathrm{hg}}^k \right)^2 - E_{\mathrm{ce}} \left(H_{\mathrm{ce}} \right)^2 \right] \Big/ 2$$

$$D^k = \left[E_{\mathrm{ce}} \left(H_{\mathrm{ce}} \right)^3 + E_{\mathrm{hg}}^k \left(H_{\mathrm{hg}}^k \right)^3 \right] \Big/ 3$$

$$N^k = H_{\mathrm{hg}}^k \sum_{i=2}^{k} \left(E_{\mathrm{hg}}^i - E_{\mathrm{hg}}^{i-1} \right) \varepsilon_0^{i-1} + \frac{\left(H_{\mathrm{hg}}^k \right)^2}{2} \sum_{i=2}^{k} \left(E_{\mathrm{hg}}^i - E_{\mathrm{hg}}^{i-1} \right) \kappa^{i-1}$$
$$+ H_{\mathrm{hg}}^k E_{\mathrm{hg}}^1 \varepsilon_{\mathrm{d}}^k + H_{\mathrm{hg}}^k \sum_{j=2}^{k} \left(E_{\mathrm{hg}}^j - E_{\mathrm{hg}}^{j-1} \right) \left(\varepsilon_{\mathrm{d}}^k - \varepsilon_{\mathrm{d}}^{j-1} \right)$$

$$Q^k = \frac{\left(H_{\mathrm{hg}}^k \right)^2}{2} \sum_{i=2}^{k} \left(E_{\mathrm{hg}}^i - E_{\mathrm{hg}}^{i-1} \right) \varepsilon_0^{i-1} + \frac{\left(H_{\mathrm{hg}}^k \right)^3}{3} \sum_{i=2}^{k} \left(E_{\mathrm{hg}}^i - E_{\mathrm{hg}}^{i-1} \right) \kappa^{i-1}$$
$$+ \frac{\left(H_{\mathrm{hg}}^k \right)^2}{2} E_{\mathrm{hg}}^1 \varepsilon_{\mathrm{d}}^k + \frac{\left(H_{\mathrm{hg}}^k \right)^2}{2} \sum_{j=2}^{k} \left(E_{\mathrm{hg}}^j - E_{\mathrm{hg}}^{j-1} \right) \left(\varepsilon_{\mathrm{d}}^k - \varepsilon_{\mathrm{d}}^{j-1} \right)$$

Supplementary Note 10. User material subroutine of dehydrated hydrogel

In order to simulate the dehydration-induced shape transformation of hydrogel, we implement the phase-evolution based constitutive model into commercial FE software packages ABAQUS (Dassault Systems, Johnston, RI, USA) by following the user material subroutine (UMAT) principle. Accordingly, we generalize the one dimensional (1D) iteration format given in Eq. (S7) into a three dimensional (3D) form

$$\Delta\boldsymbol{\sigma}^k = \boldsymbol{L}^k \Delta\boldsymbol{\varepsilon}^k - \tilde{\boldsymbol{L}}^k \Delta\varepsilon_{\mathrm{d}}^k \tag{S17}$$

where $\boldsymbol{\sigma}^k = \begin{bmatrix} \sigma_{xx}^k & \sigma_{yy}^k & \sigma_{zz}^k & \sigma_{xy}^k & \sigma_{xz}^k & \sigma_{yz}^k \end{bmatrix}^T$

$$\boldsymbol{\varepsilon}^k = \begin{bmatrix} \varepsilon_{xx}^k & \varepsilon_{yy}^k & \varepsilon_{zz}^k & \gamma_{xy}^k & \gamma_{xz}^k & \gamma_{yz}^k \end{bmatrix}^T$$

$$\boldsymbol{L}^k = \begin{bmatrix} \lambda^k + 2\mu^k & \lambda^k & \lambda^k & 0 & 0 & 0 \\ \lambda^k & \lambda^k + 2\mu^k & \lambda^k & 0 & 0 & 0 \\ \lambda^k & \lambda^k & \lambda^k + 2\mu^k & 0 & 0 & 0 \\ 0 & 0 & 0 & \mu^k & 0 & 0 \\ 0 & 0 & 0 & 0 & \mu^k & 0 \\ 0 & 0 & 0 & 0 & 0 & \mu^k \end{bmatrix}$$

$$\tilde{\boldsymbol{L}}^k = \begin{bmatrix} 3\lambda^k + 2\mu^k & 3\lambda^k + 2\mu^k & 3\lambda^k + 2\mu^k & 0 & 0 & 0 \end{bmatrix}^T.$$

Herein, the matrix $\boldsymbol{L}^k$ and vector $\tilde{\boldsymbol{L}}^k$ are defined by Lamé constants

$$\lambda^k = \frac{\left[E_{\mathrm{I}} + \left(f^{k-1} + \Delta f^k\right)\left(E_{\mathrm{U}} - E_{\mathrm{I}}\right)\right]\nu}{(1+\nu)(1-2\nu)} \tag{S18}$$

and

$$\mu^k = \frac{\left[E_{\mathrm{I}} + \left(f^{k-1} + \Delta f^k\right)\left(E_{\mathrm{U}} - E_{\mathrm{I}}\right)\right]}{2(1+\nu)} \tag{S19}$$

where $E_{\mathrm{I}} = E^0$, $E_{\mathrm{U}} = \sum_{j=0}^{n} E^j$, $f^{k-1} = \left(\sum_{j=0}^{k-1} E^j\right) \Big/ E_{\mathrm{U}}$, $\Delta f^k = E^k / E_{\mathrm{U}}$ and ν respectively represent the initial modulus, ultimate modulus, volume fraction of the first $k-1$ glassy phase, volume fraction of the kth glassy phase and Poisson's ratio.

Supplementary Note 11. Modified curved beam model for sintering ceramic

After dehydration, the ceramic layer can be treated as a curved beam of thickness H_{ce} and initial curvature κ_d. According to Fig. 4g, because the concave side of the beam is less stretched than the convex side after dehydration, shrinkage difference $\Delta\eta$ is created between the concave and convex side upon sintering. Therefore, we idealize the curved beam as a bilayer structure that compose two layers of ceramics materials with same modulus and equal thickness ($H_{ce}/2$), and the sintering-induced interlayer mismatch strain is equal to $\Delta\eta$. Based on the analysis of bilayer strip by Timoshenko (Ref. 42), we can obtain the curvature after sintering κ_s as $\kappa_s = \kappa_d - 3\Delta\eta/(2H_{ce})$.

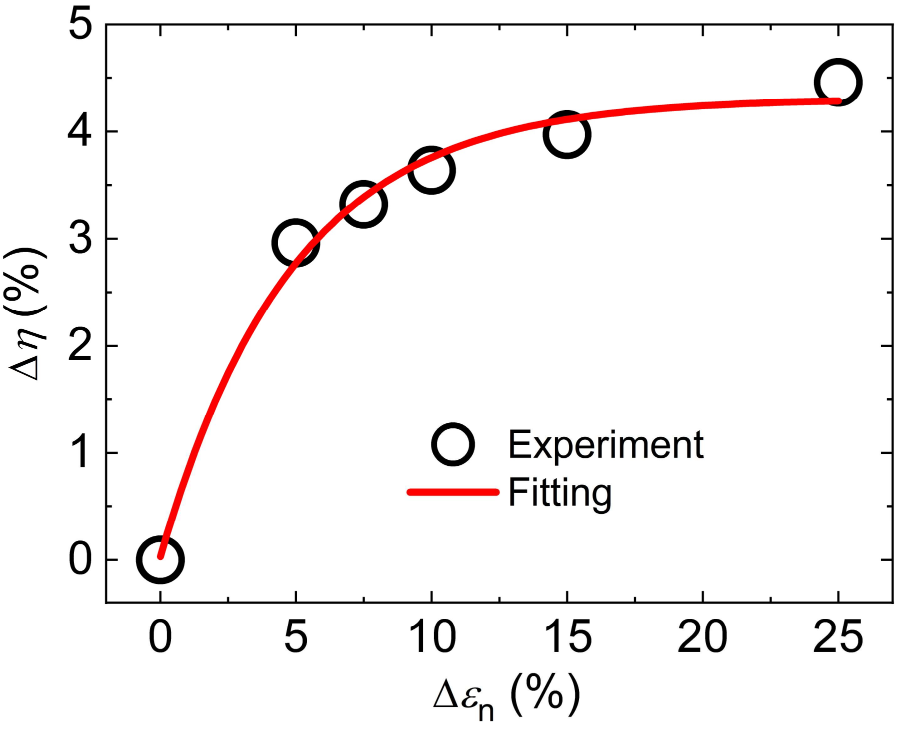

Supplementary Figure 10. Fitting result of the relationship between sintering-induced shrinkage difference Δη and normal strain difference $\Delta\varepsilon_{\mathrm{n}}$. An exponential function $\Delta\eta = C_1 \exp\left(\Delta\varepsilon_{\mathrm{n}} / C_2\right) + C_3$ is used and the fitting parameters are $C_1 = -0.0428$, $C_2 = -0.0489$ and $C_3 = 0.0431$. Source data are provided as a Source Data file.

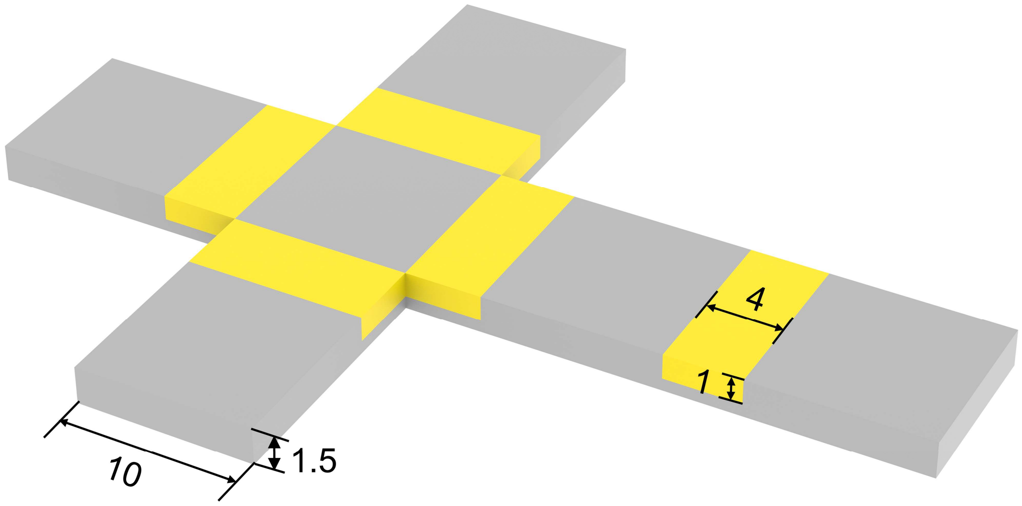

Supplementary Figure 11. Specific dimensions of flat pattern for direct 4D printing of a cube. All the dimensions are in millimeters (mm).

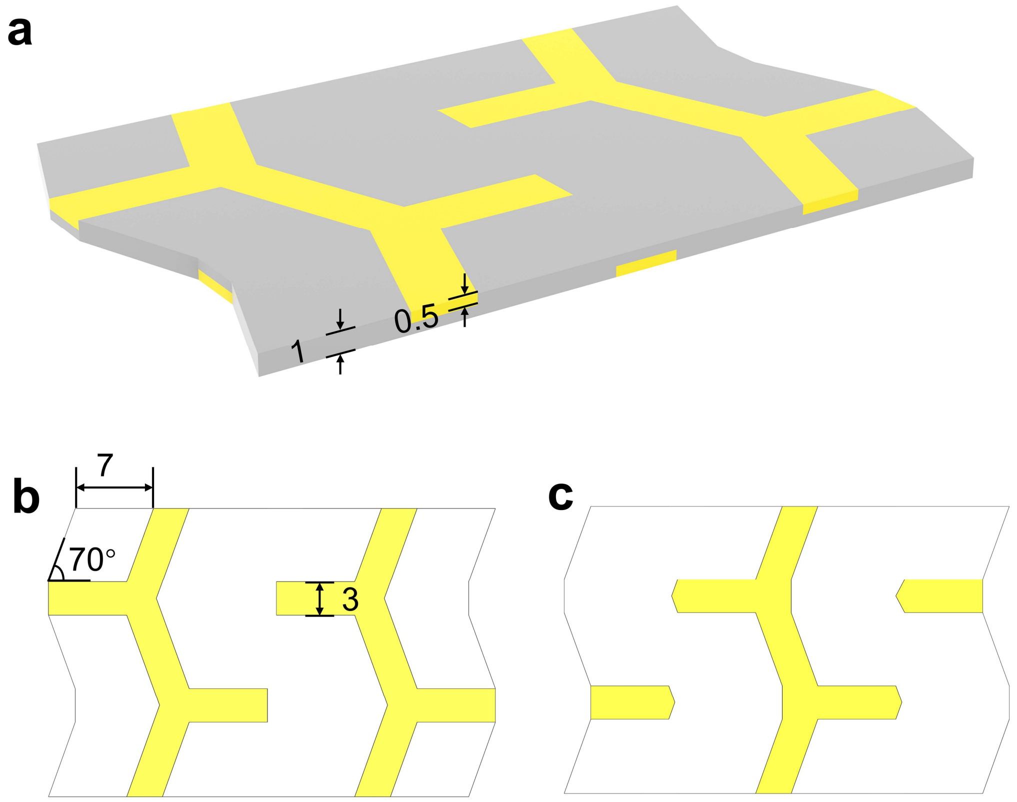

Supplementary Figure 12. Specific dimensions of flat pattern for direct 4D printing of a Miura origami. a, Perspective view. **b,** Top view. **c,** Bottom view. All the dimensions are in millimeters (mm).

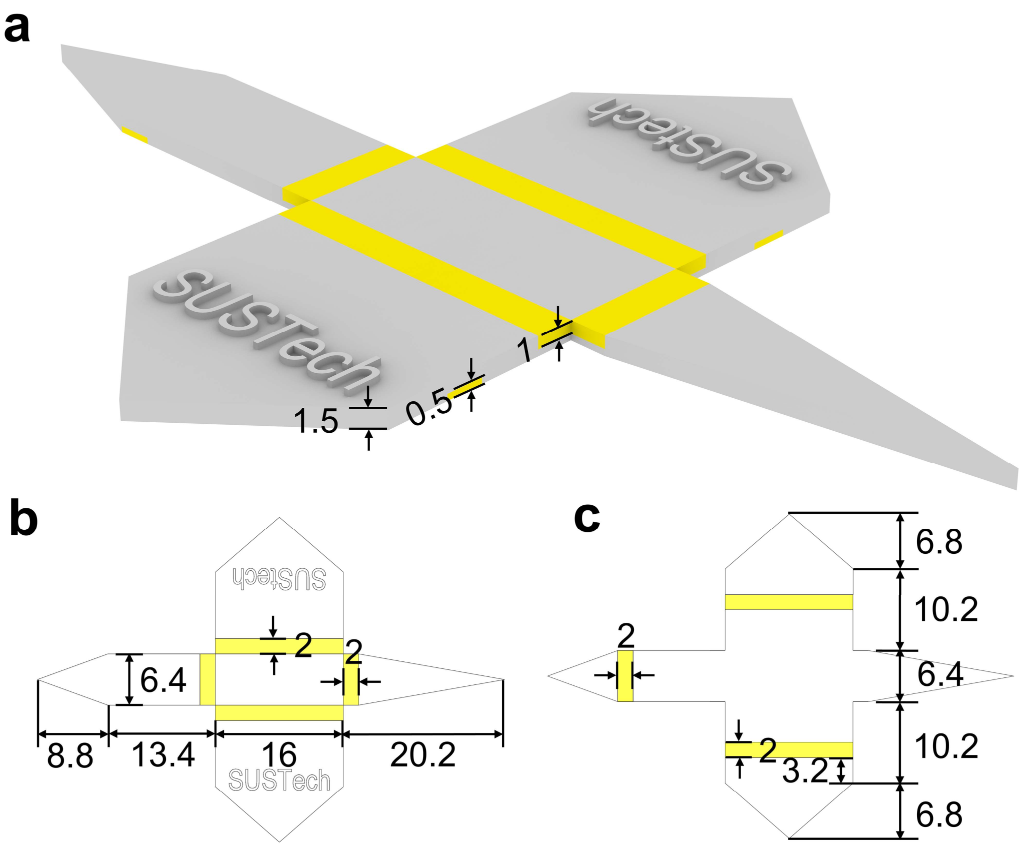

Supplementary Figure 13. Specific dimensions of flat pattern for direct 4D printing of a crane. **a,** Perspective view. **b,** Top view. **c,** Bottom view. All the dimensions are in millimeters (mm).

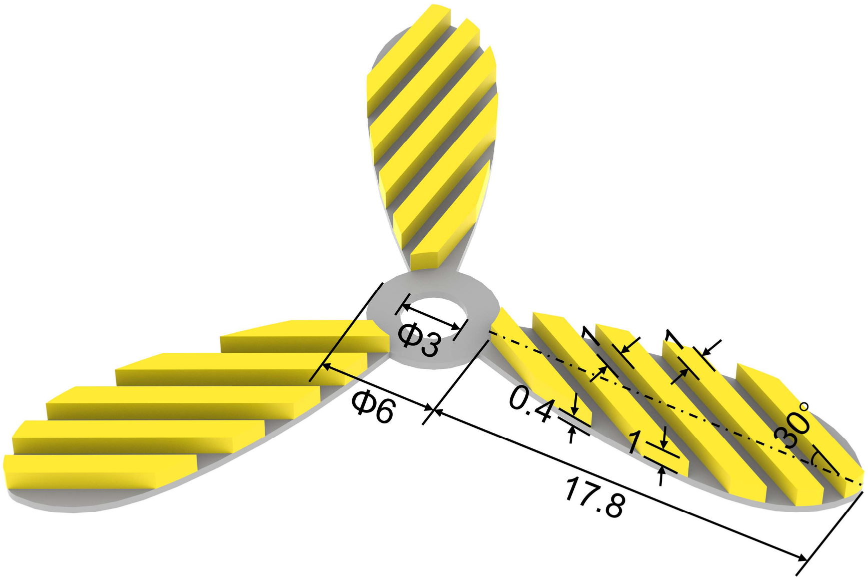

Supplementary Figure 14. Specific dimensions of flat pattern for direct 4D printing of a three-blade fan. All the dimensions are in millimeters (mm).

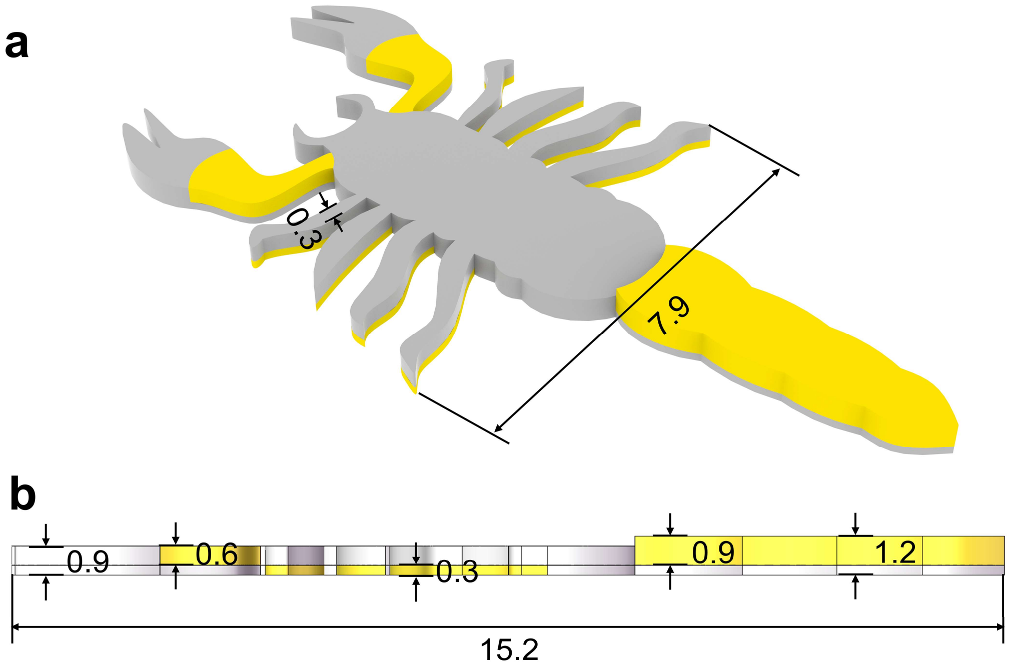

Supplementary Figure 15. Specific dimensions of flat pattern for direct 4D printing of a scorpion. a, Perspective view. **b,** Side view. All the dimensions are in millimeters (mm).
